# Histopathologic Features and Molecular Markers of Encephalocraniocutaneous Lipomatosis (ECCL)

**DOI:** 10.3390/dermatopathology12040039

**Published:** 2025-11-03

**Authors:** Siddharth Venigalla, Tanvir K. Dhaliwal, Anvita Anumolu, Lena Rafey, Arturo P. Saavedra, David D. Limbrick

**Affiliations:** 1 Department of Neurosurgery, Virginia Commonwealth University School of Medicine, Richmond, VA 23298, USA; 2 Department of Dermatology, Virginia Commonwealth University School of Medicine, Richmond, VA 23298, USAanumolua3@vcu.edu (A.A.);

**Keywords:** encephalocraniocutaneous lipomatosis, ECCL, neurocutaneous syndromes, histopathology, *FGFR1* mutation, *KRAS* mutation, molecular diagnostics, mosaic disorders

## Abstract

Encephalocraniocutaneous lipomatosis (ECCL) is a rare congenital neurocutaneous disorder characterized by ocular, skin, and central nervous system manifestations. Despite its recognizable clinical features, such as nevus psiloliparus, histopathologic characterization of ECCL remains limited in the dermatopathology literature, and diagnosis is often clinical. This scarcity of published histopathological descriptions makes diagnostic confirmation challenging and underscores the value of synthesizing the available evidence. This comprehensive review synthesizes reported histopathological findings across cutaneous manifestations highlighting key tissue-level features that may aid diagnostic confirmation. Additionally, we review the emerging role of molecular diagnostics, particularly the identification of mosaic activating mutations in *FGFR-1* and *KRAS*, which have been implicated in ECCL pathogenesis. By integrating clinicopathologic correlations with molecular insights, this review aims to enhance our dermatopathological understanding of ECCL, bolstering diagnostic reasoning and clinical decision making for this rare neurocutaneous condition.

## 1. Introduction

Encephalocraniocutaneous lipomatosis (ECCL), also referred to as Haberland syndrome, is a rare, sporadic neurocutaneous condition that is a tumor predisposition syndrome [1]. The condition is classified within the mosaic RASopathies, specifically the oculoectodermal syndrome heterogeneous groups [2,3]. ECCL is characterized by congenital anomalies affecting the central nervous system, eyes, and skin, and, to a lesser extent, the bones and heart [4]. Central nervous system manifestations that are included as part of the major criteria in diagnosis are intracranial and spinal lipomas, while ocular features may include choristomas. Cutaneous findings that are major criteria range from nevus psiloliparus, patchy or streaky non-scarring alopecia, subcutaneous lipomas in the frontotemporal region, focal skin aplasia or hypoplasia on the scalp, and small nodular skin tags on the eyelids or between the outer canthus and tragus [4]. Other findings included as major criteria are jaw tumors, multiple bone cysts, and aortic coarctation [1].

Diagnosis of ECCL primarily relies on clinical manifestations supplemented by neuroimaging studies and molecular diagnostics [1,5]. A clinical diagnosis can be made in one of the following three ways. First, an individual may have the involvement of at least three systems with major criteria in at least two body systems. Alternatively, an individual may have the involvement of at least three systems or at least one major criterion in each of two systems [1]. In each of these two cases, one major criterion is either a biopsy-proven nevus psiloliparus or a possible nevus psiloliparus with one other minor skin criterion. A molecular diagnosis can be made with suggestive clinical findings and a mosaic activating pathogenic variant in either the *FGFR1* or *KRAS* genes [1].

Treatment of ECCL focuses on managing specific disease manifestations and can involve surgical removal for symptomatic cases [5]. While no formal practice guidelines for ECCL treatment or surveillance are currently available, evaluation by a multidisciplinary team is recommended, including dermatologic exam, ophthalmologic exam, neurologic exam, brain and spinal MRI/MRA, EEG, development assessment, skeletal survey, dental evaluation, and renal ultrasound [1]. Lesions present in ECCL have a static nature and, with the exception of increased epilepsy risk, developmental delay, and intellectual disability, most individuals are able to live generally normal lives [2]. However, prognosis is unable to be reliably determined from neuroimaging findings as there is not a correlation between the extent of intracranial lesions and the severity of potential cognitive impairment, epilepsy, or skin and eye involvement [2]. Further, the initial diagnosis poses challenges as molecular testing is not always conclusive and clinical manifestations can be diverse. Various case reports and reviews have briefly touched on the fact that lipomas, angiofibromas, and fibromas may be identified on the histology of ECCL lesions [2,6]. However, a comprehensive synthesis of granular histopathological findings has yet to be performed. This scarcity of published histopathological descriptions makes diagnostic confirmation challenging and underscores the value of synthesizing the available evidence. Pathology tends to play a supportive but an underutilized role in this condition, with diagnosis based on clinical characteristics. Further, various *FGFR-1* and *KRAS* gene mutations have been linked to ECCL in prior studies [7]. Therefore, the second aim of our work is to synthesize findings on these pathogenic variants across the literature in addition to the genetic testing techniques utilized. Such findings may be crucial for advancing diagnostic precision, treatment, and the overall understanding of ECCL.

## 2. Methods

We performed a search of the PubMed and Embase databases from inception to 5 June 2025 to identify articles relevant to encephalocraniocutaneous lipomatosis. The following search queries were used across both databases: “Encephalocraniocutaneous lipomatosis” OR “ECCL” OR “Haberland syndrome” OR “nevus psiloliparus”. All case reports, case series, and reviews discussing encephalocraniocutaneous lipomatosis were eligible for inclusion. Articles were excluded if irrelevant to encephalocraniocutaneous lipomatosis. All included articles were subsequently analyzed for information pertaining to histopathologic features or molecular markers.

## 3. Results

Our search retrieved 345 unique references across both databases, of which 234 were eligible for inclusion and were reviewed for details concerning the histopathologic features and molecular markers of ECCL.

### 3.1. Histopathologic Features

#### 3.1.1. Nevus Psiloliparus

**Figure 1:**
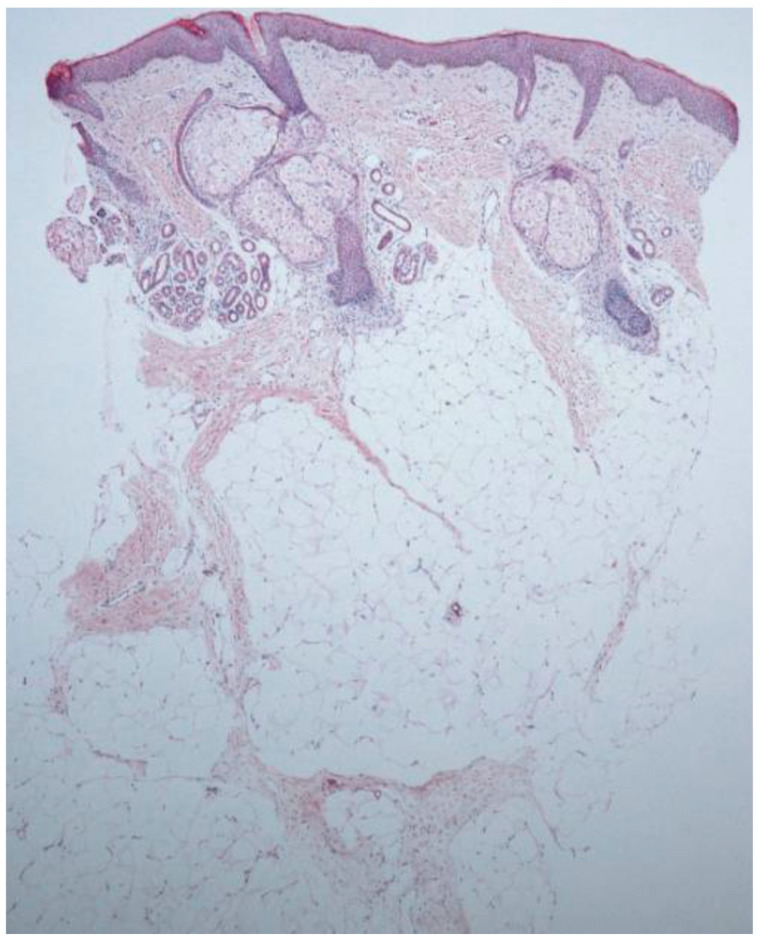
Characteristic biopsy finding of nevus psiloliparus demonstrating reduced hair follicles and lobular adipocyte proliferation. Reproduced from Figure 2A, Kim et al. (2012) [8] with permission as per the CC BY-NC 3.0 license.

The characteristic hallmark feature of ECCL is nevus psiloliparus which can be described as an alopecic plaque and a subcutaneous hamartomatous lipoma on the scalp. Histological findings of this lesion are often used to confirm diagnosis of ECCL. For example, a 1-month-old Asian male was referred for evaluation of patchy alopecia in the frontotemporal region [8]. A biopsy of the scalp showed diffuse loss of hair follicles and thickening of subcutaneous adipose tissue with lobular proliferation (Figure 1). Another case involved a 13-year-old Egyptian male who presented with an alopecic plaque on the left frontoparietal scalp, papules on the eyelid, and choristoma on the bulbar conjunctiva [9]. The histology of his scalp lesion showed the usual finding of absent hair follicles but also fibrovascular stroma. Also present were degenerated muscle fibers of the arrector pili muscles. Other reports have also identified fibrous tissue in addition to mature adipocytes in biopsies of the scalp in the temporal region [10]. This is consistent with a review of nevus psiloliparus which found increased vascularization in the dermis and capillary widening in addition to more complex hamartomatous structures, such as fibrolipomas or angiofibromas in certain regions [11]. Another 4-year-old Brazilian female presented with nevus psiloliparus, with histopathological findings of retification of the epidermis and atrophic hair follicles surrounded by densely fibrotic areas [6]. Moreover, on her biopsy they found fat extending into the upper reticular dermis as well as a virtually absent elastic fiber network. While frontal, temporal, and parietal regions are commonly the sites of nevus psiloliparus, it has also been found in the occipital region [12]. In general, findings on the histology of the scalp biopsy usually reflect increased adipose tissue, absent hair follicles, and isolated erector pili muscles. Further, nevus psiloliparus can be distinguished from other conditions, such as sebaceous nevus, due to a lack of adnexal structures on the histopathology [11].

Nevus psiloliparus has also been reported in older patients, with a 28-year-old female presenting with a scalp lesion which presented with lobules of diffuse adipocytes separated by congested capillaries [13]. While nevus psiloliparus is a frequent finding of ECCL, it is not present in all cases of the condition. For example, Happle et al. reported two cases of nevus psiloliparus that did not present with any other extracutaneous symptoms, even with a 2-year follow-up, leading them to conclude that in the future the condition may not always be considered to be a sign of ECCL [14]. Further, nevus psiloliparus can also be a feature of oculoectodermal syndrome (OES) which is another mosaic disorder [15]. The authors presented a child with fibromas on the eyelids and epibulbar dermoid, a scalp lesion that showed a paucity of hair follicles, and linearly arranged arrector pili muscles in the midcorium with suspected fat protrusion of the reticular dermis on the histopathology, leading them to conclude that it was nevus psiloliparus. However, based on the clinical features, the authors still concluded a diagnosis of OES.

#### 3.1.2. Vascular Hyperplasia and Inflammatory Infiltrates

**Figure 2:**
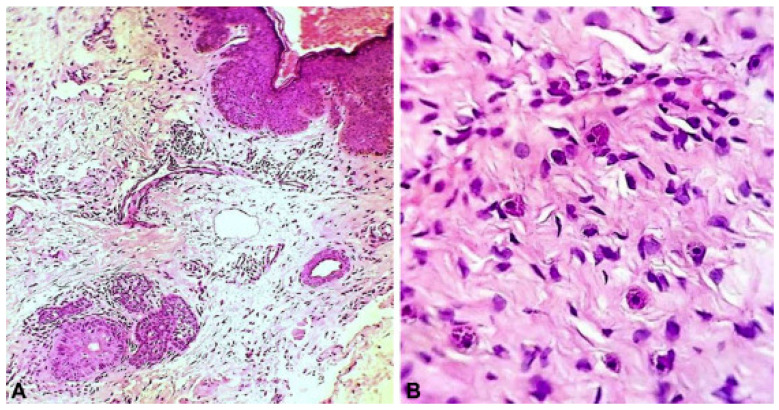
Eyelid papule demonstrating (**A**) dermal fibrosis and vascularization along with (**B**) inflammatory infiltrate. Reproduced from Alakad et al. (2015) [9] with permission as per the CC BY-NC-ND 4.0 license.

The histopathology of papules on the scalp and the face in one of the early cases of ECCL showed irregularly shaped collagen fibers in the dermis with abnormally small diameters [16]. Further, they found fat extending into the reticular dermis as well as a diffuse inflammatory infiltrate containing a large number of mast cells. A sample of the papule on the temple showed dermal fibrosis arranged in a lamellar array along with vascular hyperplasia. The vascular hyperplasia reported in the papules was consistent with the case reported by Alakad et al., suggesting that this may be a common theme among papules found in ECCL [9]. Further, that patient also presented with a biopsy of the eyelid papules that showed both fibrovascular stroma and vascular hyperplasia, as well as inflammatory infiltrate (Figure 2). Another case of encephalocraniocutaneous lipomatosis accompanied by odontomas found fibromas with vascularized stroma on a biopsy of the facial papules present in a 7-year-old male [17]. Aside from vascular hyperplasia, inflammatory infiltrate has also been reported in the dermis of facial lesions in those with ECCL. For example, a 14-year-old male with ECCL and otolaryngologic involvement displayed large inflammatory cells infiltrated with irregular collagen fibers and an increase in fibroblast number in the reticular layer of a postauricular lesion [18]. Other findings of such facial papules have also included broad bands of hyperelastic tissue fibers and clumps of fragmented elastic tissue fibers mixed with collagen bundles, suggesting a connective tissue nevus of elastic tissue type [6].

Adipose tissue may also be present in the dermis of facial lesions in those with ECCL alongside fibrous connective tissue. For example, a biopsy of a temple papule in a 1-month-old Filipino male revealed clusters of adipocytes within the dermis that were bordered by bundles of collagen fibers and fibrosis [19]. Connective tissue nevi have also been reported in ECCL with one study confirming this finding on a biopsy of a mass at the lateral canthus of the left eye [20]. Another case of a 5-year-old male presented with a nodular skin tag of the lateral canthus of his right eye [21]. The histopathology of this nodular skin tag showed disorganized elements of fibrous and adipose tissue, leading the authors to conclude that it was a lipomatous hamartoma. Another nodular skin tag in a 5-year-old female presenting with seizures also showed a hamartoma with disorganized elements of fibrous tissue and fat [22]. Vascular hyperplasia has also been reported in such skin tags, as two skin tags from the cheeks of a 16-year-old female who was referred for severe alopecia were identified as angiofibromas on histologic evaluation [23]. In a comparison of oculocerebrocutaneous syndrome (OCCS) and ECCL, the authors reviewed skin findings of ECCL and noted skin tags in 57% of the cases with inconsistent pathology [24]. These lesions included a neurofibroma, connective tissue nevi, normal epidermis with sebaceous hyperplasia, one lesion with normal epidermis, fat and cartilage, one containing collagen and elastic tissue, and a vascular fibroma. A summary of the cutaneous histological findings of ECCL is presented in Table 1.

### 3.2. Molecular Markers and Pathways

Two major genes have been cited by several studies as being associated with ECCL: *KRAS* and *FGFR-1*. Both genes have been linked to somatic postzygotic mosaicism. To date, molecular diagnostics are used to confirm a diagnosis of ECCL off the clinical guidelines offered by Moog et al. with two of the three of major central nervous system, ocular, or skin manifestations present [1]. Further, it has been suggested that mosaicism is easier to detect based on gene testing of affected skin tissues, particularly the fibroblast culture overlying the nevus psiloliparus.

Exome sequencing of five individuals with ECCL demonstrated two mosaic mutations: c.1638C > A (p.Asn546Lys) and c.1966A > G (p.Lys656Glu) in the tyrosine kinase domain of *FGFR1* [7]. These mutations were each in two individuals with allele frequencies of 33% and 42% for p.Asn546Lys and 45% and 47% for p.Lys656Glu. To detect these mutations, in addition to exome sequencing, single molecular inversion probes (smMIPs) were used, which have increased the ability to detect low frequency mosaic mutations which may not have been captured by Sanger sequencing. DNA derived from the fibroblasts provided a higher diagnostic yield than those from blood, buccal, or saliva samples. This suggests that affected scalp tissue does indeed provide the best measure of the mosaic genetic mutations of ECCL. The authors noted that these have also been identified as oncogenic mutations in the literature which may explain the increased risk for low grade gliomas in patients with ECCL. In another patient with ECCL and a pilocytic astrocytoma, the authors identified a N546K mutation in *FGFR-1* which showed a differential distribution across all samples including those unaffected tissues [25]. The authors also highlighted the use of the droplet digital polymerase chain reaction (ddPCR) technique for detecting low levels of autosomal mutations in blood or swabs. Various point mutations in the *FGFR-1* gene have been reported including an *FGFR1* p.K656E variant using next generation sequencing [26]. These mutations, specifically *FGFR1* K656E and *FGFR1* N546K, were also found in a whole exome sequencing of five ECCL-associated brain tumors [27]. The authors also noted coexisting mutations in *FGFR1/RAS/MAPK* pathway genes, including *NF1, KRAS*, *PTPN11*, and *FGFR1* mutations. The *KRAS* gene has also been linked to the pathogenesis of ECCL. In a review of patients with mosaic RASopathies including ECCL, the majority of ECCL patients had mutations in the *KRAS* gene, with one patient presenting with an *NRAS* gene [28].

It is possible for multiple major clinical features of ECCL to be present with negative genetic testing for the *FGFR* and *KRAS* genes. A recent case of ECCL was reported where a 50-day-old female newborn presented with biopsy-confirmed nevus psiloliparus and two intracranial lipomas on a brain MRI [29]. However, a somatic nevus gene testing panel using next generation sequencing came back negative for mutations in the *FGFR1* or *KRAS* genes. In fact, in another case of a newborn who experienced left-sided lesion in the brain, arachnoid cysts, and optic nerve colobomas, the authors performed newborn genetic screening, chromosomal analysis, microarray, *FGFR* related disorders via the *FGFR-1* gene, and a comprehensive brain malformation sequencing panel, which all returned negative [30]. Another patient presented with Wilms tumor and nevus psiloliparus as well as lipomas in the left cerebellopontine angle and the spinal cord came back negative for *FGFR* Sanger analysis of the peripheral blood [31]. This further underscores the need for genetic testing of affected tissues with a focus on *RAS* pathway genes which provide the highest yield.

Recent studies suggest that dysregulated signaling pathways involving progranulin and angiogenesis may contribute to the pathogenesis of ECCL as it is involved in cutaneous and systemic manifestations. ECCL is not only structural, regarding *FGFR1* and *KRAS* mutations, but also may be influenced by dysregulated pathways that are common to other proliferative conditions. Progranulin, a secreted glycoprotein with pleiotropic functions, is a mediator of angiogenesis, inflammation, and tissue remodeling in both tumoral and non-tumoral settings [32]. In visceral adipose tissue, progranulin exerts progangiogenic effects by stimulating endothelial cell proliferation and migration, in part through upregulation of vascular endothelial growth factor (VEGF) and activation of downstream pathways such as PI3K/AKT [32]. By promoting endothelial cell proliferation, migration, and neovascularization, progranulin contributes to aberrant vascular remodeling, a hallmark that may overlap with the vascular malformations and lipomatous overgrowths seen in ECCL. The findings of Binișor et al. underscore progranulin’s role in associating adipose tissue expansion with angiogenic activity, suggesting that similar mechanisms may underlie the dysregulated vascular and connective tissue development clinically seen in ECCL. Therefore, aberrant activation of VEGF-PI3K/AKT pathways by progranulin may represent one of the molecular drivers of ECCL pathology, linking histopathologic features with dysregulated molecular signaling pathways.

#### Oculoectodermal Syndrome (OES)

Oculoectodermal syndrome (OES) also shares several clinical characteristics with ECCL: focal alopecia, epibulbar dermoids, upper eyelid lesions, and linear hyperpigmentation. Further, both conditions have been identified as mosaic disorders and have been linked to *KRAS* mutations. In a study of four patients, three with OES and one with ECCL, four *KRAS* mutations were identified: c.437C > T (p.Ala146Val), c.436G > A (p.Ala146Thr), c.437C > T (p.Ala146Val), and c.436G > A (p.Ala146Thr) [15]. Most of these positive mutation results were obtained from fibroblast tissues which were similar to previous findings in ECCL. This further corroborates the use of these affected skin tissues for genetic testing for mosaic conditions such as ECCL and OES. OES/ECCL mutations often feature mutations of Gly-13, Leu-19, and Ala-146 to Asp-13, Thr-146, and Phe-19. The introduction of negatively charged aspartate and bulky residues alter the GTP binding properties of the domain which have been hypothesized as the mechanism for the mosaic effects of these mutations. Various other mosaic RASopathies, such as nevus sebaceus and non-organoid keratinocytic epidermal, have been linked to other genes, like *HRAS*. *KRAS* mutations (such as *KRAS* c.436G > A, p.(Ala146Thr)) have also been linked to other mosaic disorders, such as Schimmelpenning–Feuerstein–Mims syndrome [33]. One group even detected a c.436G > A (p.Ala146Thr) *KRAS* mutation in an ECCL patient using high throughput sequencing and Sanger sequencing of DNA extracted from the patient’s scalp lipoma [34]. It is important to note that this variant was not detected by Sanger sequencing in the patient’s blood, saliva, buccal swab, or fibroblasts cultured from a scalp biopsy.

Some researchers have considered OES a milder subtype of ECCL, viewing the conditions as part of a spectrum rather than two distinct entities. One example is Richters et al. who reported on a 3-year-old male presenting with a congenital hairless area with bullae covering it and a lipodermoid cyst and diagnosed him with OES/ECCL [35]. The authors performed both Sanger sequencing and used a single molecular inversion probe (smMIP) to look for mutations in the *FGFR-1* among other genes. They found no pathogenic variant, exon duplication, or deletion in the *FGFR-1* gene. However, their smMIP analysis found *NRAS* variant c.182A > G (p.(Gln61Arg)) at a 16% allele frequency in the affected skin tissue. Azrak et al. reported the second case of ECCL with an *NRAS* mutation, which involved a female infant with a c.37G > C (p.Gly13Arg) variant at 35.6% allele frequency and an associated intracranial lipoma [36]. These two cases further suggest that *NRAS* mutations may also be present in the spectrum of mosaic disorders including ECCL and OES in addition to the more frequently reported *FGFR-1* and *KRAS* genes. Additionally, due to the presence of mesenchymal tumors and defects in vasculogenesis present in patients with ECCL, it has also been hypothesized that a mutation in a gene similar to the transcription factor HMG2A may be involved [4]. Another recent case identified a germline variant in the *NF1* gene in a patient with an ECCL phenotype, leading the authors to infer that ECCL may be part of a spectrum of conditions associated with *NF1* variants [37]. A summary of the reported variants across genes and testing techniques utilized is presented in Table 2.

## 4. Conclusions and Future Directions

Encephalocraniocutaneous lipomatosis is a rare condition with ocular, cutaneous, and neurological manifestations. In this review, we have consolidated the histopathologic features across its cutaneous findings. In addition, we have also compiled all genetic variants linked to ECCL in the literature and provided an overview of various molecular diagnostics utilized. The benchmark for diagnosis of ECCL is based on the criteria by Moog which requires two of the three major features to be present, those being nevus psiloliparus, ocular choristomas, and intracranial/intraspinal lipomas [4]. This summary of the histopathological features and molecular markers may guide clinicians who are on the edge of making a diagnosis or further assist diagnostic confirmation if multiple major features are present.

**Figure 3:**
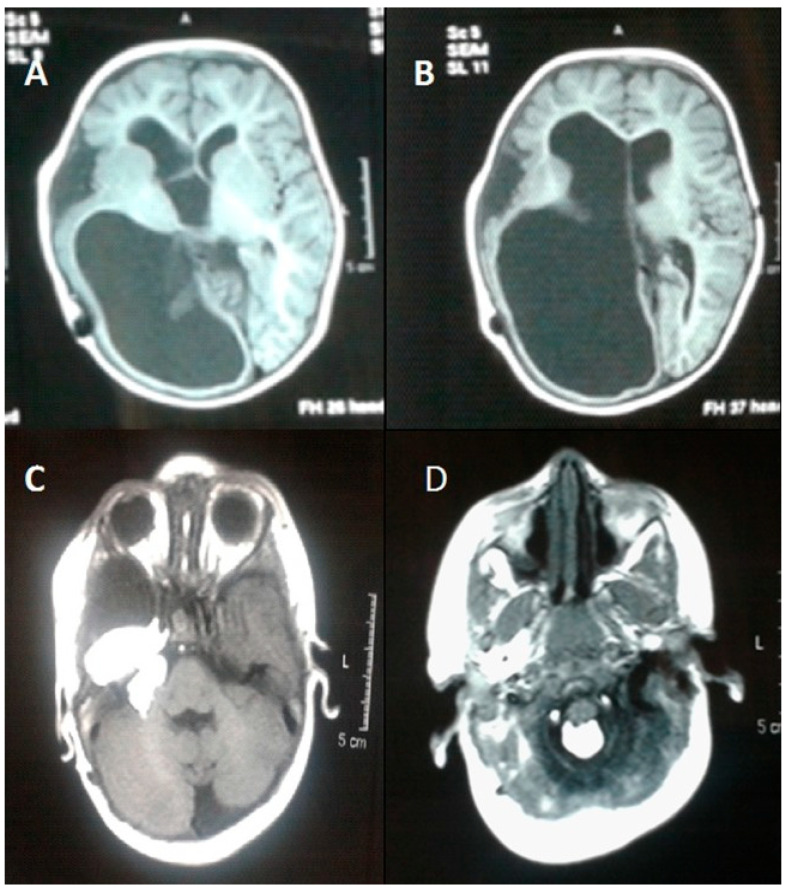
A representation of CNS features associated with ECCL via T-1 weighted magnetic resonance imaging of the brain. (**A**) Intravenous contrast showing, (**B**) dilation of the lateral ventricle and atrophy of the right cerebral hemisphere with prominence of the overlying subarachnoid spaces, (**C**) a fat containing mass measured at 25 × 12 mm visible at the right cerebellopontine cistern angle, and (**D**) similar fatty lesions in the posterior aspect of the magnum foramen and cervical canal. Reproduced from Naous et al. (2015) [39] with permission as per the CC BY-NC 3.0 license.

Encephalocraniocutaneous lipomatosis has multiple other manifestations aside from the skin, including choristomas in the eyes and intracranial lipomas and intraspinal lipomas (Figure 3) [38,39]. The histology of these lesions could be further explored in the literature to be used in conjunction with histological findings of the skin to aid diagnostic reasoning. For example, some eye lesions have been noted as epibulbar dermoids with findings of lacrimal gland, cartilage, and adipose tissue covered by nonkeratinized, stratified squamous epithelium [40]. The histopathology of CNS lesions can vary from lipomas to even reported cases of an intracranial sarcoma [41]. Further consolidation of the reported histopathology of eye and CNS lesions could be helpful to clinicians treating patients with suspected diagnosis of ECCL.

Our review bridges the gap between dermatopathology and molecular genetics to enhance diagnostic reasoning in this rare but recognizable condition. Although diagnosis is typically made on clinical features, the scarcity of published histopathological descriptions limits pathologists’ and clinicians’ ability to recognize tissue-level patterns with confidence. As a result, pathology remains supportive but underutilized in practice despite its potential to refine and confirm clinical impressions. Systematic case collection with standardized dermatopathology reporting, coupled with the integration of molecular findings, would enable stronger correlations between clinical and pathological findings and allow for cross-institutional comparison. This would not only improve diagnostic accuracy in this rare but clinically recognizable condition but also reinforce the role of histopathology as a valuable adjunct to molecular testing in understanding ECCL pathogenesis.

ECCL requires a multilayered evaluation to improve diagnostic accuracy and to understand the local manifestations of this systemic disease. While individual reports have described features such as adipose infiltration, vascular hyperplasia, fibrosis, and connective tissue nevi, systematic histopathologic and immunohistochemical evaluation of ECCL lesions is still lacking. Insights from more common systemic conditions where integrated evaluation has refined diagnostic accuracy may clarify the relationship between systemic pathology and local tissue changes. For example, Fărcaş-Berechet et al. highlighted that in patients with diabetes mellitus, associating clinical findings with histological and immunohistochemical studies of periodontal tissues revealed insights into local disease manifestations [42]. A similar systematic approach remains undeveloped in ECCL. Applying a similar framework to ECCL may provide a more standardized basis for the diagnosis and interpretation of cutaneous and extracutaneous lesions. Moreover, it may also reveal how mosaic genetic alterations translate into the tissue-level changes observed in ECCL.

## Figures and Tables

**Table 1:** Clinical features of ECCL along with their reported histopathological features.

Clinical Cutaneous Findings	Reported Associated Histopathological Features
Alopecic scalp lesion	Absent hair follicles, lobular fat infiltration of the dermis, and isolated arrector pilli muscles [8] Fibrovascular stroma [9] Degenerated muscle fibers of arrector pilli muscles [9] Potential for complex hamartomous structures such as fibrolipomas or angiolipomas [10] Retification of the epidermis and hair follicles surrounded by fibrosis and absent elastin fiber network [6] Lack of adnexal structures [11] Irregularly shaped collagen fibers in the dermis [16] Inflammatory infiltrate including mast cells [16] Diffuse adipocytes separated by congested capillaries [13]
Facial and eyelid papules	Fibrovascular stroma [9,17] Vascular hyperplasia [9,16] Inflammatory infiltrate [9,18] Dermal fibrosis in a lamellar array [16] Connective tissue nevi including bands of hyperelastic tissue fibers mixed with collagen bundles [6,20] Cluster of adipocytes in the dermis bordered by bundles of collagen fibers and fibrosis [19,21,22]
Skin tags	Hamartoma with disorganized elements of fibrous tissue and fat [21,22] Vascular hyperplasia [23]

**Table 2:** Reported genetic variants associated with ECCL and sequencing techniques used.

Gene.	Pathogenic Variant	Sequencing Technique Used
*FGFR-1*	p.Asn546Lys [7,25,27] p.Lys656Glu [7,26,27]	Single molecular inversion probes (smMIPs) [7] Exome Sequencing [7,27] Droplet digital polymerase chain reaction (ddPCR) technique [25] Sanger sequencing [7,25] Next generation sequencing [26]
*KRAS*	p.Ala146Val [15] p.Ala146Thr [15,34]	Sanger sequencing [15,34] High throughput sequencing [34]
*NF1*	K2375N [27] (c.5234C>G; p.(Ser1745*)) [37] (c.3916C>T; p.(Arg1306*)) [37]	Whole exome sequencing [27] Sanger sequencing [37]
*PTPN11*	E69K [27]	Whole exome sequencing [27]
*NRAS*	p.(Gln61Arg). [35] p.Gly13Arg [36]	Single molecular inversion probe (smMIP) [35] Next generation sequencing [36]

## Data Availability

Not applicable.
